# Daily artificial intelligence-assisted adaptive small margin radiotherapy during trimodal therapy of muscle-invasive bladder cancer

**DOI:** 10.3389/fonc.2026.1729810

**Published:** 2026-02-12

**Authors:** Philip Blumenfeld, Daniel Levy, Yair Hillman, Jon Feldman, Marcel Fang, Ayman Salhab, Ofer N. Gofrit, Aron Popovtzer, Marc Wygoda

**Affiliations:** 1 Department of Radiation Oncology, Sharett Institute of Oncology, Hadassah Hebrew University Hospital, Jerusalem, Israel; 2 Department of Urology, Hadassah Hebrew University Hospital, Jerusalem, Israel

**Keywords:** adaptive radiotherapy (ART), artificial-intelligence, bladder preservation, cone-beam C-arm CT, margin reduction, muscle-invasive bladder cancer

## Abstract

**Objectives:**

Adaptive radiotherapy (ART) enables daily plan modification based on anatomy and may improve outcomes in muscle-invasive bladder cancer (MIBC) treated with bladder-preserving chemoradiation. This retrospective study evaluated the dosimetric and clinical outcomes of daily ART.

**Methods:**

Between March 2021 and August 2024, 37 patients with T2–T4a MIBC received 55 Gy in 20 fractions with or without chemotherapy. Patients were stratified by margin strategy: small-margin (SM, n=26) adaptive versus scheduled plans, and small-margin adaptive versus large-margin (SM-LM, n=11) scheduled plans. Daily cone beam CT–guided ART with AI-assisted contouring, physician review, and plan selection was performed. Planning target volumes (PTVs) were generated by expanding the clinical target volume (CTV) by 0.5–1.0 cm in SM plans; 1.0–1.5 cm in LM plans. Toxicity was graded using CTCAE v5.0.

**Results:**

Across 740 fractions, adaptive plans were chosen in 96.9%. ART improved coverage in both cohorts. In SM patients, mean PTV V95 increased from 92.9% to 99.3% and CTV V98 from 96.7% to 99.8% (p < 0.01). In SM-LM comparison, PTV V95 improved from 95.5% to 99.8% (p < 0.01), with rectum and bowel dose reductions up to 91.9% and 57.8% (rectum V100%, bowel V100% respectively, both p < 0.01). At 12 months, cystectomy-free, progression-free, and overall survival were 95.8%, 82.7%, and 92.8%. No grade ≥3 acute toxicities occurred.

**Conclusions:**

Daily ART improves target coverage, reduces organ-at-risk exposure, and enables margin reduction in bladder-preserving radiotherapy. This represents one of the largest series of daily online ART for MIBC, demonstrating that margin-reduced ART is feasible and improves dosimetry compared with non-adaptive workflows.

## Introduction

Bladder preservation treatment in patients with T2-4a bladder cancer, includes maximal transurethral resection of bladder tumor (TURBT) followed by chemoradiotherapy. It is an established alternative to immediate radical cystectomy in selected patients with muscle- invasive bladder cancer (MIBC) (1, 2). Conformal radiotherapy for bladder cancer presents significant challenges due to the variability in bladder and rectal filling, which results in substantial interfraction changes in bladder shape and position (3–5). To compensate, large anisotropic planning target volume (PTV) margins (up to 2 cm) are traditionally employed, often exposing nearby organs at risk (OARs), such as the rectum and bowel, to high-dose radiation (6).

Adaptive radiotherapy (ART) enables clinicians to respond to these anatomical changes by modifying the treatment plan based on daily imaging (7). This process accounts for changes in the target, organ motion, and daily volume variations, potentially improving both tumor targeting and normal tissues sparing (8). The advent of on-board Cone Beam Computed Tomography (CBCT) and automated adaptive planning systems has allowed the implementation of online ART workflows, which facilitate daily plan adaptation based on patient-specific anatomy (9).

In the pelvis, the need for ART is particularly significant. Daily bladder and rectal volume fluctuations result in large dose variations, which might compromise target coverage and increase OAR toxicity if not appropriately managed (10–12). By reducing the need for large PTV margins, ART may allow tighter dose distributions, preserving tumor control while reducing toxicity. Recently, there has been growing interest in adaptive radiotherapy approaches for bladder cancer, particularly for CBCT guided adaptation. Several studies have demonstrated the feasibility of such approaches, including the geometric and dosimetric benefits over conventional image-guided radiotherapy (13–16). However, few studies have evaluated fully online daily CBCT-based ART workflows in routine clinical practice, with available data on comprehensive dosimetric assessment and early clinical outcomes remaining limited.

This study aimed to quantify the dosimetric advantages of daily ART and to assess its potential clinical impact in patients with MIBC undergoing bladder-preserving chemoradiation. We hypothesized that daily ART would provide superior target coverage and enhanced OAR sparing compared to scheduled (non-adaptive) planning approaches, consequently being associated with a lower toxicity profile, without compromising the oncological outcomes.

## Methods

### Patient selection and treatment

All patients with clinical stage T2–T4a muscle-invasive bladder cancer (MIBC), including select cases with regional lymph node involvement (N1) and oligometastatic disease (M1), treated between March 2021 and August 2024, were evaluated through a multidisciplinary uro-oncology clinic. Initial assessment included a physical exam, laboratory testing (CBC, liver and kidney function), and imaging with either contrast-enhanced CT Chest, Abdomen and Pelvis or FDG PET-CT. Histopathological confirmation via cystoscopy was obtained in all cases.

Patients who were either medically unfit for radical cystectomy or elected for bladder preservation underwent maximal TURBT followed by radiation with or without chemotherapy. Eligibility criteria included preserved bladder function, absence of significant hydroureteronephrosis (HUN), and willingness to undergo regular cystoscopic surveillance. Neoadjuvant and concurrent chemotherapy were administered at the discretion of the treating physician.

The study was conducted in accordance with the Declaration of Helsinki (as revised in 2013) and approved by the institutional review board (protocol #0238‐17‐HMO). The requirement for written informed consent was waived owing to the retrospective nature of the analysis. This study is reported in accordance with the STROBE (Strengthening the Reporting of Observational Studies in Epidemiology) guidelines.

### Adaptive radiotherapy workflow

**Figure 1:**
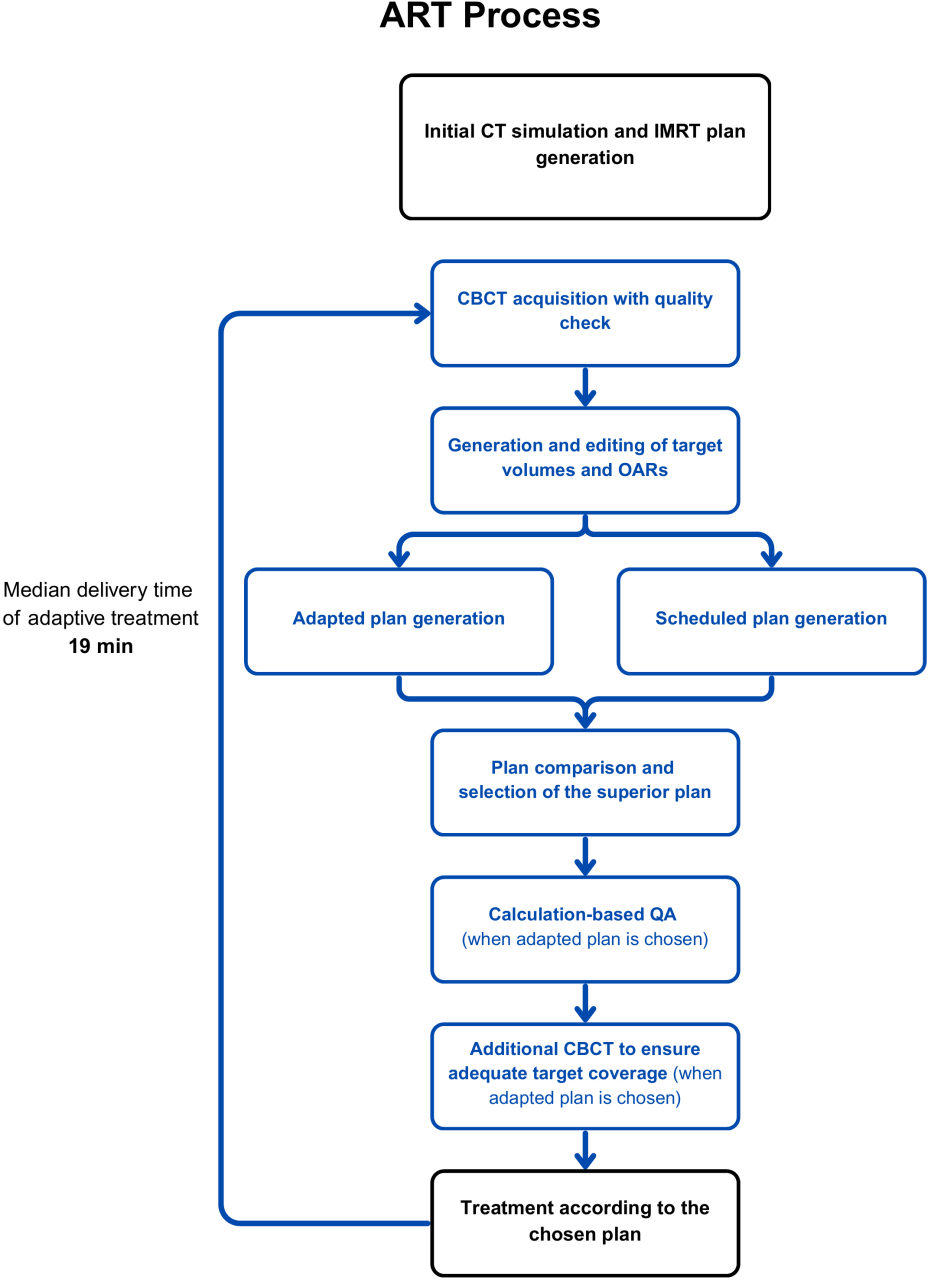
Adaptive radiotherapy workflow.

Daily online ART was performed using Varian Ethos Treatment Unit Version 1.1 and Varian Ethos Treatment Management Version 2.1 (Varian Medical Systems, Palo Alto, CA, US). (the ART workflow is illustrated in **Figure 1**). Prior to treatment, every patient underwent a CT simulation, followed by IMRT plan generation. Each treatment session included pre-treatment CBCT, auto-contouring via deformation of structures, Artificial Intelligence (AI) contouring, and manual contour approval by the radiation oncologist. Ethos treatment planning uses auto-segmentation based on AI for a predetermined set of influencer structures (structures that influence the shape of common clinical targets). In this case, the influencer structures created via auto-segmentation include the rectum, bladder, and bowel. These influencer structures are detected automatically on CBCT imaging by use of AI algorithms based on a convolutional neural network.

Synthetic CTs were generated via deformable registration of the initial simulation CT, and the scheduled (simulation-based) plan was calculated on them. The scheduled plan refers to the original simulation-based treatment plan created prior to therapy and applied across all fractions without re-optimization. For the scheduled plan the Ethos system calculates an optimal couch shift based on a fusion of the simulation CT and the CBCT and the updated daily target positions. Simultaneously an adaptive (CBCT-based) plan was optimized based on the updated daily anatomy, and compared to the scheduled plan. The superior plan was selected for treatment at the discretion of the treating radiation oncologist. Independent dose calculation–based quality assurance (QA) was performed using Mobius3D and MobiusAdapt (Varian Medical Systems) on the first treatment fraction and subsequently at the discretion of the treating team. Additionally, a verification CBCT was performed when a patient had been on the couch for more than 10 minutes.

### Simulation and target volumes

CT simulation was performed in the supine position using standard pelvic immobilization, including a knee support and foot fixation to enhance setup reproducibility. Patients were aligned using external laser marks and daily CBCT-based image guidance. Axial slice thickness was ≤3 mm. Patients were instructed to void prior to simulation and each treatment. The clinical target volume (CTV) included the whole bladder and proximal urethra. When clinically indicated (n = 5), pelvic nodal irradiation was performed. The nodal clinical target volume (CTVn) included the internal and external iliac, obturator, perivesical, and presacral nodal regions, contoured according to institutional practice based on published pelvic nodal atlases. A nodal planning target volume (PTVn) was generated using a uniform 0.7 cm expansion. Organs at risk (OARs) contoured included the rectum (from anal verge to rectosigmoid junction), bowel loops (represented by the term “bowel”), and femoral heads.

### Planning cohorts

To reflect evolving clinical practice, patients were stratified into two cohorts based on margin strategy applied to the scheduled (non-adaptive) plans. Margin selection for scheduled plans was determined on a case-by-case basis at the discretion of the treating physician, reflecting real-world clinical practice in the absence of daily online adaptation. At first, both scheduled and adaptive plans employed small margins. As experience with adaptive radiotherapy increased, larger margins were applied to scheduled plans to ensure target coverage and to allow a clearer evaluation of the true dosimetric benefit of margin-reduced adaptive planning. This resulted in the SM-LM cohort, in which adaptive plans used small margins while scheduled plans used larger margins. Cohort assignment was determined by the treatment period. Patients treated between March 2021 to October 2023 were included in the SM cohort while patients treated between November 2023 to August 2024 are in the SM-LM cohort, following the institutional change in margin policy:

- SM cohort (n=26): Both adaptive and scheduled plans used small-margin (SM) planning target volumes (PTVs) (0.5–1.0 cm).
- SM-LM cohort (n=11): Adaptive plans used SM PTVs, while scheduled plans used large-margin (LM) PTVs (1.5 cm anterior/superior, 1.0 cm posterior, 0.8 cm elsewhere).

In the SM cohort, margins within the 0.5–1.0 cm range were selected based on treating physician experience and institutional familiarity with daily online adaptive radiotherapy. Earlier treatments employed more conservative margins, which were progressively reduced as experience with the adaptive workflow increased and consistent target coverage was demonstrated.

All patients were prescribed a dose of 55 Gy in 20 fractions to the PTV and 44 Gy in 20 fractions to the PTVn (when nodal irradiation was performed).

### Endpoints and statistical analysis

Primary endpoints included dosimetric comparisons between adaptive and scheduled plans within each cohort. These included PTV V95%, CTV V98%, CTV D98%, and rectal and bowel volumes receiving 1.24 Gy to 2.75 Gy (corresponding to rectal V45%, V75%, V91%, V100% and bowel V68%, V75%, V91%, V100%). The metrics used for OAR evaluation represent institutional constraints based on the RAIDER trial (17). CTV metrics are reported as evaluative study endpoints and were not used as planning objectives. Additionally, CTV D98% is reported as a study endpoint for comparative evaluation of scheduled and adapted plans and does not represent a guideline-based CTV coverage recommendation.

Clinical endpoints included acute (within 3 months post-treatment) and late (beyond 3 months) genitourinary (GU) and gastrointestinal (GI) toxicities, as well as cystectomy-free survival (CFS), progression-free survival (PFS), and overall survival (OS). Toxicity was assessed weekly using CTCAE v5.0. Follow-up included cystoscopy and imaging every 3–6 months.

Statistical analyses were performed using paired t-tests or Wilcoxon signed-rank tests, with significance set at p < 0.05. Kaplan-Meier curves were used to estimate CFS, PFS, and OS.

## Results

A total of 37 patients with a median age of 73 years (range 48–89) were treated with daily ART. Thirty-six had urothelial carcinoma (TCC) and one had basaloid squamous cell carcinoma (BSCC). Most patients (84.8%) presented with stage II disease, and the majority (89.2%) received concurrent chemoradiotherapy. Neoadjuvant chemotherapy was administered in 35.1% of cases, and 10.8% received adjuvant immunotherapy. Patient and treatment characteristics are summarized in **Table 1**.

**Table 1:** Patient and treatment characteristics.

Characteristic	N (%) or Median (Range)
Sex	
Male	33 (89.2%)
Female	4 (10.8%)
Age	
	Median 73 (Range 48-89)
T stage	
T1	1 (2.7%)
T2	29 (78.4%)
T3	5 (13.5%)
T4	2 (5.4%)
N stage	
N0	32 (86.5%)
N1	2 (5.4%)
N2	1 (2.7%)
N3	2 (5.4%)
M stage	
M0	36 (97.3%)
M1	1 (2.7%)
Group stage	
Stage II	31 (84.8%)
Stage III	5 (13.5%)
Stage IV	1 (2.7%)
Treatment	
Chemoradiotherapy	33 (89.2%)
Radiation alone	4 (10.8%)
Neoadjuvant chemotherapy	13 (35.1%)
Adjuvant immunotherapy	4 (10.8%)
Histological subtype	
TCC	36 (97.3%)
BSCC	1 (2.7%)

A total of 740 comparative adaptive and scheduled plans were generated for 37 patients. The adaptive plan was selected in 96.9% of fractions. The median delivery time of an adaptive treatment was 19.4 minutes (from CBCT acquisition to completion of treatment). The average daily PTV volume change was 14.1% (range 0% - 157.3%).

**Table 2** summarizes the dosimetric comparison of target coverage metrics between adaptive and scheduled plans. In the SM cohort, adaptive planning resulted in a significant improvement in target coverage, primarily reflected by higher mean PTV V95% compared to scheduled plans (99.3% *vs*. 92.9%, p < 0.01). This increase was accompanied by consistent improvements across additional target coverage metrics with adaptation including mean CTV D98% (99.9% *vs* 98.0%) and mean CTV V98% (99.8% *vs*. 96.7%) compared to scheduled plans, both p < 0.01. In the SM cohort, none of the patients required re-contouring or re-optimization of the plan following the verification CBCT (when performed).

**Table 2:** Adaptive versus scheduled (non-adapted) target dosimetric comparison.

Cohort	Target	Fractions evaluated	Mean daily adapted plan coverage (SD)	Mean daily scheduled plan coverage (SD)	Mean daily difference adapted versus scheduled (95% CI)	P-value
*SM cohort*	*Mean PTV55 V95*	520	99.3% (0.9%)	92.9% (9.3%)	6.4% (5.7% - 7.2%)	< 0.01
	*Mean CTV55 V98*	520	99.8% (0.5%)	96.7% (9.5%)	3.1% (2.3% - 4.0%)	< 0.01
	*Mean CTV55 D98*	520	99.9% (1.0%)	98.0% (6.8%)	1.9% (1.3% - 2.5%)	< 0.01
*SM-LM cohort*	*Mean PTV55 V95*	180*	99.8% (0.3%)	95.5% (5.8%)	4.3% (3.5% - 5.1%)	< 0.01
	*Mean CTV55 V98*	220	100.0% (0.0%)	99.2% (2.0%)	0.8% (0.6% - 1.1%)	< 0.01
	*Mean CTV55 D98*	220	100.8% (0.9%)	99.4% (2.4%)	1.4% (1.1% - 1.7%)	< 0.01

*PTV data were unavailable for 40 sessions, preventing evaluation in those cases.

In the SM-LM cohort, dosimetric outcomes were consistent with those observed in the SM-LM cohort. Adaptive planning achieved significantly higher mean PTV V95% when compared with scheduled plans (99.8% *vs*. 95.5%, p < 0.01). Corresponding improvements with adaptation were also noted in the supporting coverage metrics, including CTV D98% (100.8% *vs*. 99.4%), as well as CTV V98% (100.0% *vs*. 99.2%) compared to scheduled plans, both p < 0.01.

**Figure 2:**
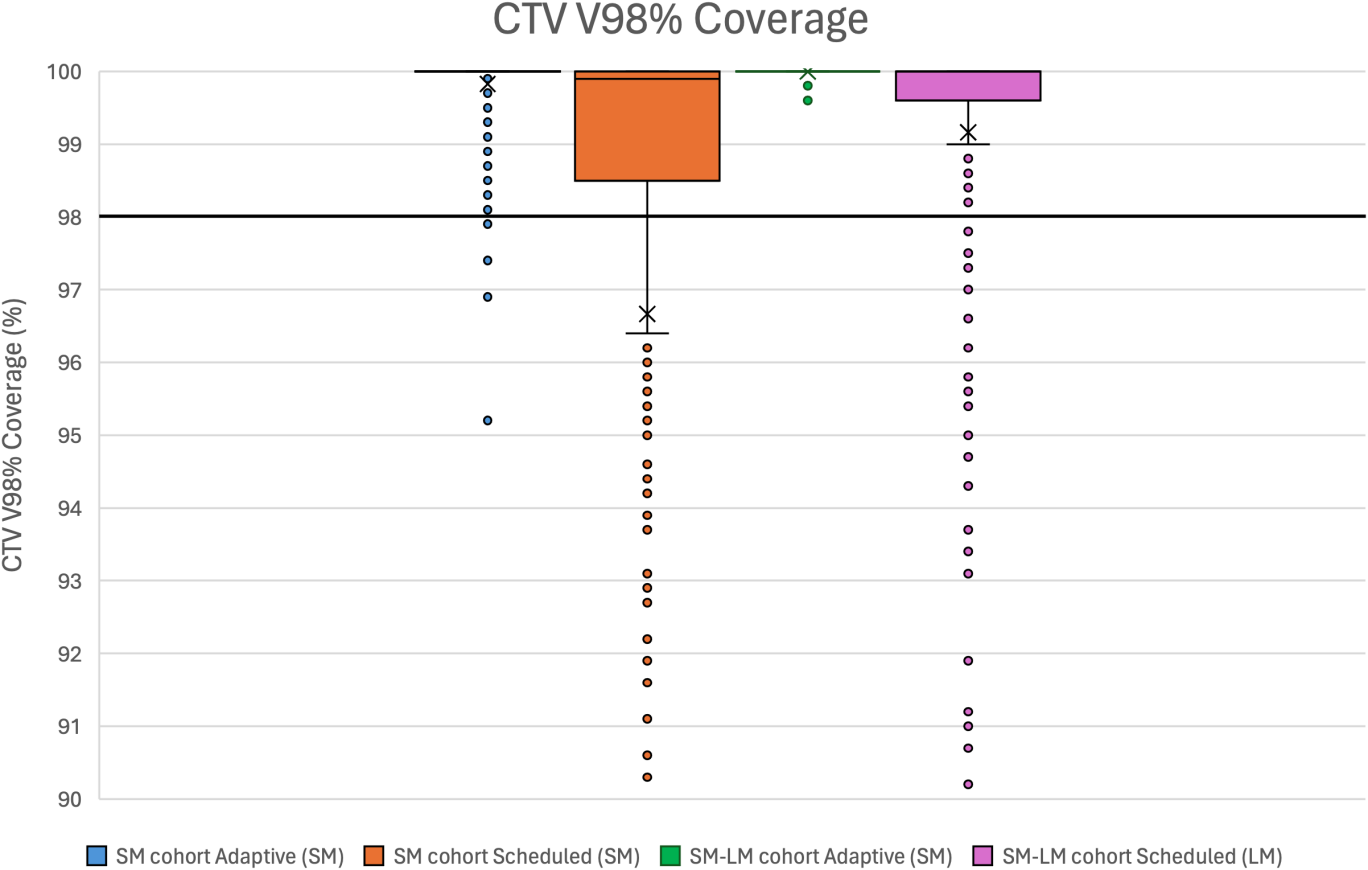
CTV V98% coverage across cohorts with adaptive and scheduled strategies.

**Figure 2** displays the CTV V98% coverage across all 740 evaluated fractions. Scheduled non-adaptive plans demonstrated considerable variability and inconsistency in target coverage. The greatest variability was observed in the scheduled SM plans (SM cohort) (SD = 9.5%), where 115 of 520 fractions (22%) had inadequate CTV coverage (CTV V98% < 98%). Scheduled LM plans (SM-LM cohort) (SD = 2.0%) showed an improvement, but 30 of 220 fractions (14%) failed to meet the 98% CTV V98% goal. In contrast, adaptive planning achieved consistent CTV coverage. In the SM cohort, only 5 of 520 adaptive plans (1%) fell below 98% (SD = 0.5%), while in the SM-LM cohort, all 220 adaptive fractions achieved CTV V98% above 99.5% (SD = 0.0%).

**Table 3** summarizes the dosimetric differences in OAR exposure between scheduled and adaptive plans. In the SM cohort, when comparing scheduled SM plans to adaptive SM plans, adaptive planning resulted in a 5.9% relative reduction in rectal V45% (p<0.01). However, rectal exposure at higher doses (V75%, V91%, V100%) showed no significant reduction and instead increased with adaptive planning by 3.5% (p = 0.5), 23.9% (p < 0.01), and 55.1% (p < 0.01), respectively. Changes in bowel dose exposure were minor and inconsistent across dose levels. In the SM-LM cohort, when comparing adaptive SM plans to scheduled LM plans, adaptive planning resulted in reductions in rectal volume receiving 2.75 Gy (V100%) by 91.9%, 2.50 Gy (V91%) by 89.8%, 2.06 Gy (V75%) by 82.2%, and 1.24 Gy (V45%) by 66.5% (all p < 0.01). Similarly, bowel volume receiving 2.75 Gy, 2.50 Gy, 2.06 Gy, and 1.87 Gy was reduced by 57.8%, 56.5%, 26.1%, and 20.0%, respectively (all p < 0.01).

**Table 3:** Adaptive versus scheduled organ at risk sparing comparison per session.

Cohort	Organ at risk	Dose received (%)	Fractions evaluated	Mean difference in irradiated volume % [Scheduled – Adaptive] (95% CI)	P-value	Relative reduction with adaptive
*SM cohort*	*Rectum*	1.24 Gy (45%)	520	1.4% (0.4% - 2.4%)	< 0.01	5.9%
		2.06 Gy (75%)	520	-0.1% (-0.6% - 0.4%)	0.5	-3.5%
		2.50 Gy (91%)	520	-0.5% (-0.8% - (-0.1%))	< 0.01	-23.9%
		2.75 Gy (100%)	520	-0.5% (-0.7% - (-0.2%))	< 0.01	-55.1%
	*Bowel*	1.87 Gy (68%)	520	-0.1% (-0.5% - 0.3%)	0.5	-1.2%
		2.06 Gy (75%)	520	-0.1% (-0.4% - 0.1%)	0.3	-2.2
		2.50 Gy (91%)	520	-0.0% (-0.2% - 0.2%)	0.9	-0.2%
		2.75 Gy (100%)	520	0.1% (-0.1% - 0.2%)	0.3	5.6%
*SM-LM cohort*	*Rectum*	1.24 Gy (45%)	220	20.2% (18.0% - 22.9%)	< 0.01	66.5%
		2.06 Gy (75%)	220	4.9% (4.0% - 5.8%)	< 0.01	82.2%
		2.50 Gy (91%)	220	2.3% (1.8% - 2.8%)	< 0.01	89.8%
		2.75 Gy (100%)	220	0.9% (0.7% - 1.2%)	< 0.01	91.9%
	*Bowel*	1.87 Gy (68%)	220	2.8% (2.1% - 3.4%)	< 0.01	20.0%
		2.06 Gy (75%)	220	2.8% (2.3% - 3.4%)	< 0.01	26.1%
		2.50 Gy (91%)	220	3.2% (2.7% - 3.7%)	< 0.01	56.5%
		2.75 Gy (100%)	220	2.0% (1.6% - 2.4%)	< 0.01	57.8%

**Figure 3:**
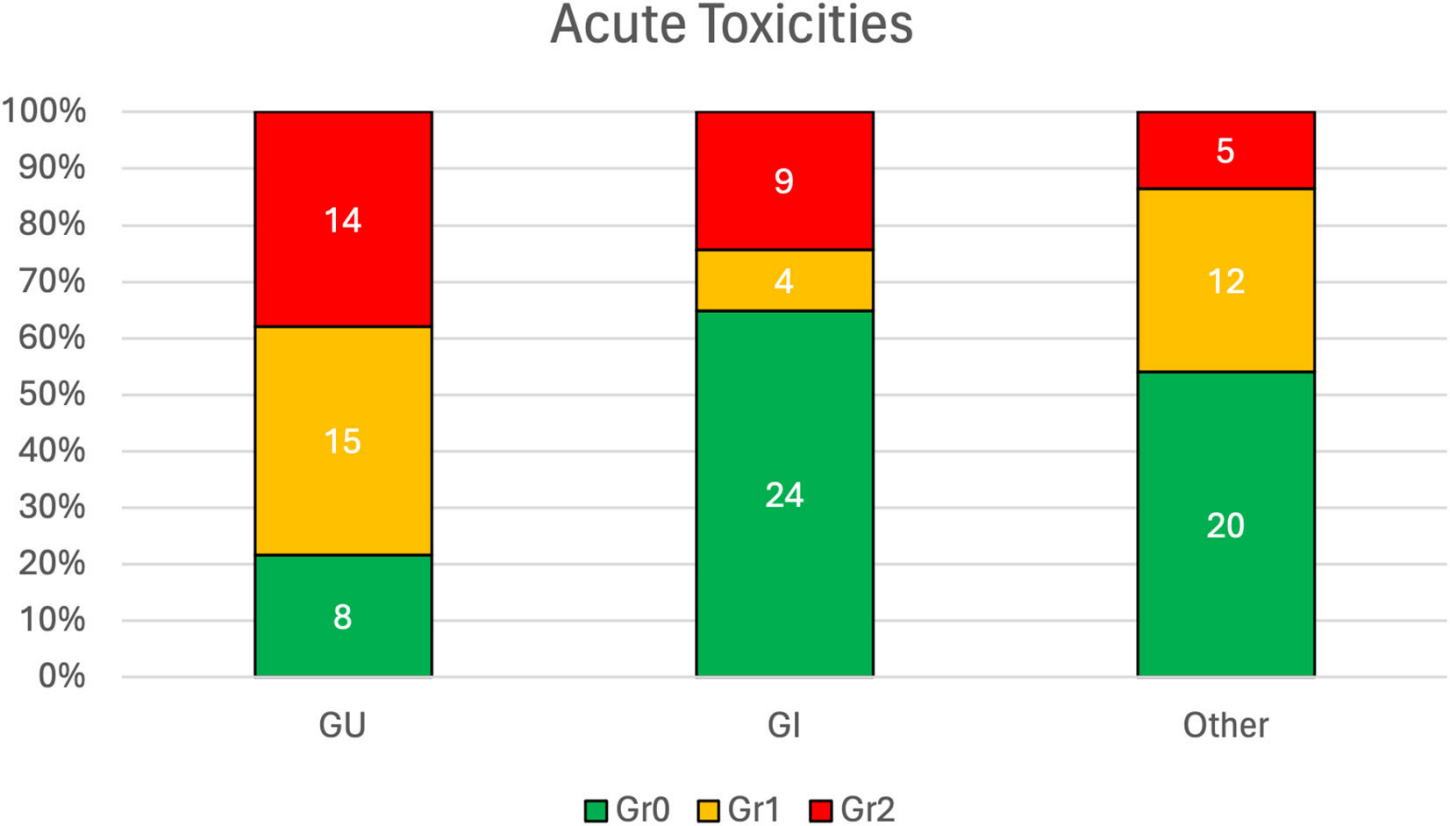
Acute toxicities (CTCAE Gr0–Gr5) for GU, GI, and other toxicities.

**Figure 4:**
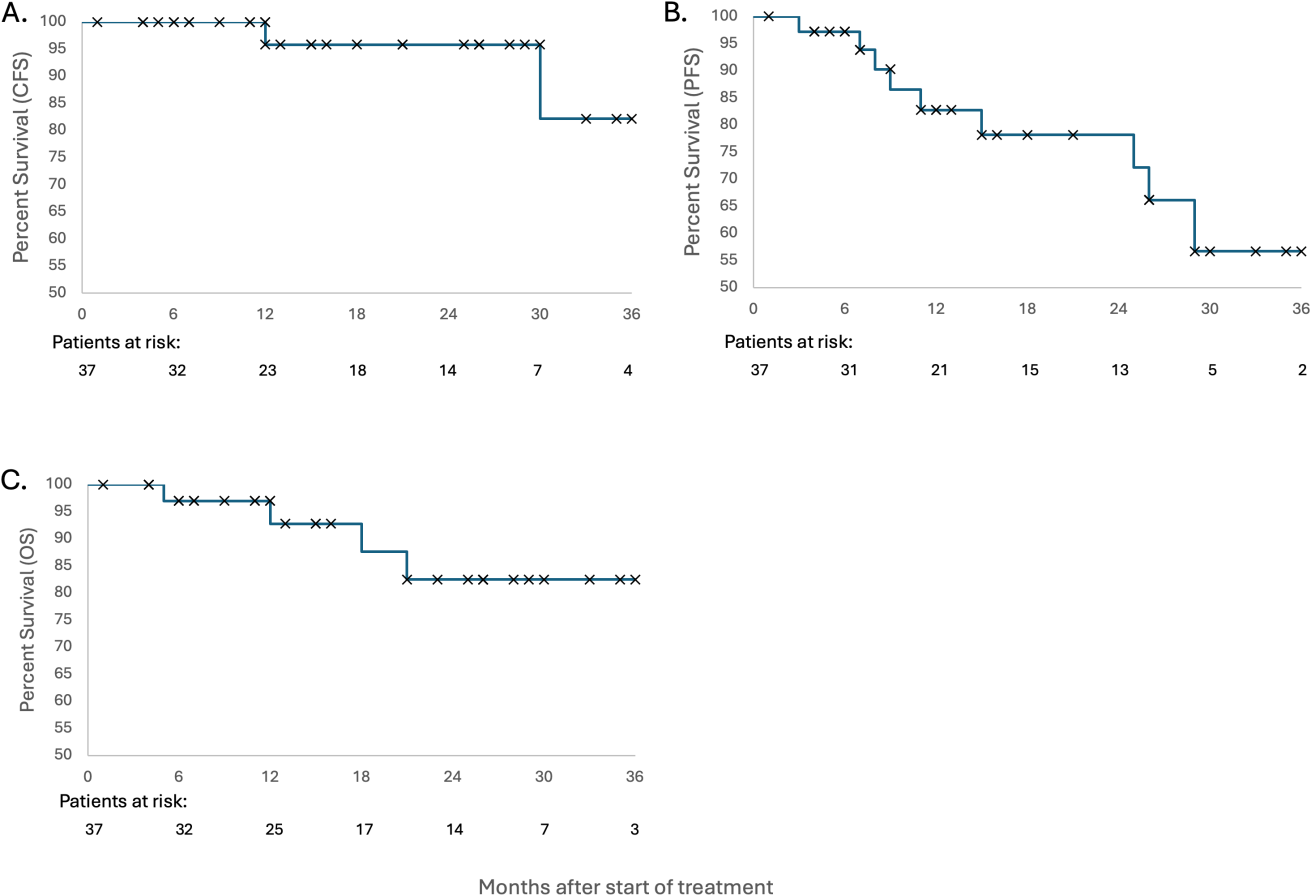
Kaplan-Meier curves for CFS **(A)**, PFS **(B)**, and OS **(C)**.

See **Figure 3** for acute and long-term toxicities. At a median follow-up of 16 months, only grade 1–2 acute toxicities were reported, and long-term grade 3 radiation cystitis occurred in 2 patients (5.4%). See **Figure 4** for long-term treatment outcomes. Among all patients (n = 37), the 1-year CFS, PFS, and OS rates were 95.8%, 82.7%, 92.8%, respectively.

## Discussion

Daily online adaptive radiotherapy (ART) offers a novel strategy for bladder-preserving chemoradiation by addressing interfraction anatomical variability (3, 14, 18). In this study, adaptive plans were selected in 96.9% of 740 fractions, with a median treatment time of 19.4 minutes. The average daily PTV volume change was 14.1% (range, 0–157.3%), emphasizing the need for daily plan adaptation. Patients were stratified into two cohorts based on margin strategy: in the SM cohort (n=26), both adaptive and scheduled plans used small margins, while in the SM-LM cohort (n=11), adaptive plans used small margins and scheduled plans used large margins.

ART significantly improved target coverage in both cohorts. In the SM cohort (small margin adaptive *vs* small margin scheduled), adaptive planning corrected for anatomical variations, improving PTV V95 from 92.9% to 99.3% and CTV V98 from 96.7% to 99.8% (p < 0.01). When performed, the verification CBCT demonstrated sufficient PTV coverage, requiring no further modification, thus indicating the feasibility of the small PTV margins to provide clinically acceptable target coverage. However, this improvement in coverage sometimes resulted in modest increases in OAR exposure, as adapting small-margin plans could bring more rectum or bowel into the high-dose region to maintain target coverage. In contrast, the SM-LM cohort (small margin adaptive *vs* large margin scheduled) better reflects real-world non-adaptive practice, where large margins are needed to maintain target coverage. In this comparison, adaptive small-margin planning not only substantially reduced rectal and bowel dose (reductions exceeding 50% in high-dose volumes) but also achieved slightly better target coverage (PTV V95 improved from 95.5% to 99.8%, and CTV V98 from 99.2% to 100.0%, all p < 0.01). These findings demonstrate that the full dosimetric advantage of ART, both in optimizing target coverage and minimizing OAR exposure, is realized when comparing margin-reduced adaptive planning to conventional large-margin non-adaptive workflows.

Moreover, ART reduced variability in dose delivery, as evidenced by lower standard deviations across all evaluated metrics. This pattern was observed in both cohorts and suggests more consistent and reliable dose delivery with ART. While the mean CTV V98% in the SM-scheduled cohort was acceptable overall, closer examination revealed significant inconsistencies. As shown in **Figure 2**, 115 of 520 fractions (22%) had unacceptable CTV coverage (CTV V98% < 98%) with scheduled plans. This demonstrates that margin reduction without daily adaptation is not reliably feasible in conventional image-guided workflows. In the SM-LM cohort, scheduled plans using large margins achieved acceptable CTV coverage in most fractions but at the cost of increased OAR doses, and 30 of 220 fractions (14%) still fell below the 98% threshold. In contrast, adaptive plans demonstrated the least variability, with only 5 of 740 fractions falling below 98% coverage, and all fractions maintaining CTV V98% ≥ 95.2%.

Prior studies have highlighted the potential of ART in bladder cancer, although most relied on offline or hybrid approaches, adapting only a subset of fractions (14, 19, 20). In contrast, our study utilized a fully online adaptive workflow for every treatment fraction, allowing consistent plan refinement and individualized dose delivery throughout the course. Fischer et al. reported improved rectal and bowel sparing using CBCT-guided ART, but their implementation was limited to selected fractions (14). Similarly, Kong et al. showed the feasibility of online re-optimization using CBCT, though primarily in early clinical settings without comprehensive outcome reporting (19). Our data support and extend these findings, demonstrating that the dosimetric advantages of ART are most pronounced when used daily with reduced margins, a design that closely mirrors modern clinical application. Our findings reinforce the concept that ART’s principal benefit lies in margin reduction. Several studies have reported mixed or minimal OAR benefit when adaptive and non-adaptive plans used similar margins (8, 21). By contrast, our dual-cohort design allowed for direct comparison between small-margin ART and traditional large-margin scheduled plans, revealing dramatic reductions in rectal and bowel dose. These results align with the conclusions of Bondar et al. who suggested that margin reduction is the key mechanism by which ART enhances safety without compromising efficacy (10). Åström et al. recently demonstrated the feasibility and dosimetric benefits of online ART using a platform using CBCT for bladder cancer. Their experience, though based on fewer patients and a mixed ART/non-ART protocol, supports our findings regarding PTV reduction and OAR sparing, with median rectal V50Gy reduced by 70.7% and bowel V45Gy by 18.8% compared to non-adaptive plans (16). These outcomes mirror our observations and further validate the advantage of daily re-optimization to the anatomy of the day.

In the HYBRID trial, Huddart et al. assessed an adaptive plan-of-the-day (PoD) strategy in a frail, elderly population receiving ultra-hypofractionated therapy. Although this approach relied on pre-generated plans rather than online re-optimization, it demonstrated significantly lower acute grade ≥3 non-GU toxicity (6% *vs*. 13%) and an encouraging 3-month local control rate of 81% (22). While our population differed in age and treatment paradigm, the clinical benefits observed with adaptation remain consistent and support ART’s broader utility across patient groups and fractionation schemes. The potential of ART across tumor sites is increasingly being recognized. Shelley et al. implemented daily CBCT-guided ART using Ethos for cervical cancer, reporting significant reductions in bowel, bladder, and rectum D2cc per fraction, as well as improvements in PTV D98% (23). While anatomical and motion patterns differ between bladder and cervical tumors, their findings reinforce the broader dosimetric and clinical feasibility of AI-driven ART using the same platform. Daily ART also allowed us to account for substantial day-to-day variability in bladder and PTV volumes, with an average 14.1% change per fraction. Contrary to some reports suggesting progressive bladder filling during treatment, we observed no consistent longitudinal trends (24), emphasizing the need for daily imaging and adaptation rather than reliance on baseline or mid-treatment planning (25).

Clinically, our outcomes compare favorably to historical data and to our own institutional experience on bladder preservation (1, 2). After a year of follow up, we observed a cystectomy-free survival of 95.8%, progression-free survival of 82.7%, and overall survival of 92.8%. Our results further support the viability of bladder preserving chemoradiation. Notably, no grade ≥3 acute toxicities were reported, and only 5.4% of patients developed late grade 3 radiation cystitis. These outcomes compare favorably with those reported in historical studies using conventional planning (26, 27), further underscoring the benefit of daily ART. Our study has several limitations. As a retrospective single-institution analysis, it is subject to selection bias and lacks randomization. We could not control for all anatomical and clinical covariates, and although this is one of the largest reported cohorts using daily online ART for MIBC, our patient-level sample size remains modest for assessing clinical and oncological outcomes. In addition, while we used two different margin strategies to reflect evolving clinical practice, this introduced heterogeneity into our comparative analysis. Finally, our study was not powered to evaluate long-term outcomes or cost-effectiveness, which should be addressed in future prospective trials.

In summary, our findings provide compelling real-world evidence that daily ART for bladder cancer enhances target coverage, significantly reduces radiation to normal tissues, and enables safe margin reduction. With high feasibility, favorable toxicity, and promising early oncologic outcomes, daily ART represents a powerful tool in advancing bladder-preserving treatment. Future multicenter studies and long-term follow-up will be essential to validate these results and support broader implementation.

## Data Availability

The raw data supporting the conclusions of this article will be made available by the authors, without undue reservation.
